# Evaluation of metabolic changes in FDG PET/CT imaging after mRNA-based COVID-19 vaccination

**DOI:** 10.55730/1300-0144.5519

**Published:** 2022-07-09

**Authors:** Serkan İŞGÖREN, Gizem KÖROĞLU, Gözde DAĞLIÖZ GÖRÜR, Hakan DEMİR

**Affiliations:** Department of Nuclear Medicine Kocaeli University Faculty of Medicine, Kocaeli, Turkey

**Keywords:** COVID-19, vaccines, positron emission tomography, messenger RNA

## Abstract

**Background/aim:**

It is important to evaluate the vaccine-related metabolic changes on FDG PET/CT to avoid confusing results. We here aimed to assess the frequency and intensity of regional and systemic metabolic PET/CT changes of patients who received the mRNA-based COVID-19 vaccine (BNT162b2-Pfizer/BioNTech) and to analyze possible factors affecting these changes.

**Materials and methods:**

Among the patients who underwent FDG PET/CT for any indication in our department between July 2021 and December 2021, 129 volunteer patients with a history of COVID-19 vaccination were included in this prospective observational study. Bilateral axillary lymph nodes, ipsilateral deltoid muscle, bone marrow, spleen, thyroid, and liver FDG uptakes were evaluated visually and semiquantitatively for each examination.

**Results:**

The frequencies of positive axillary lymph nodes after vaccination were 40%, 44.4%, 32.6%, and 44.7% in all, 1st dose, 2nd dose, and heterologous vaccination regimens groups, respectively. Maximum standardized uptake values of spleen, liver, and bone marrow were statistically high in patients with positive axillary lymph nodes than with negative ones (p < 0.05). Positive deltoid muscle uptake and diffusely increased thyroid uptake findings were observed in 10 and 8 patients, respectively. The median time interval between vaccination and imaging was 9.5 days for patients with positive axillary lymph nodes and 17 days for patients with negative nodes. In our study group, only 8 patients had a positive documented history of COVID-19 infection.

**Conclusion:**

Regional and systemic metabolic changes were occasionally found on FDG PET/CT imaging in patients who received the mRNA-based COVID-19 vaccine. To avoid these timely decreasing changes, we recommend managing the ideal timing of imaging or vaccination and taking a careful history.

## 1. Introduction

Mass vaccination against SARS-CoV-2 is considered to be the main solution to control the COVID-19 pandemic [1]. Mass vaccination in Turkey was started in early 2021 and two different vaccine products (CoronaVac-Sinovac and BNT162b2-Pfizer/BioNTech) are currently in use. With the development of more-immunogenic mRNA vaccines and the widespread vaccine application, the increase of vaccine-related metabolic changes in F-fluorodeoxyglucose (FDG) PET/CT imaging will be seen more commonly [2–7]. Among these metabolic changes, regional hypermetabolic lymphadenopathy in the axilla has become an increasingly common finding and there are several studies recognizing COVID-19 vaccine-associated lymphadenopathy [8–13]. This also poses a challenge for medical imagers in patients with malignancies prone to axillary node dissemination. Therefore, it is crucial for medical imagers to recognize this potential imaging pitfall as an incidental finding in oncological settings to avoid overdiagnosis. It is important to recognize the frequency of immune activation-induced changes in recently vaccinated patients and their association with variables in FDG PET/CT studies.

In this study, we aimed to explore the incidence of metabolic PET/CT changes in patients who received the mRNA-based COVID-19 vaccine (BNT162b2-Pfizer/BioNTech) and to evaluate the factors affecting these changes.

## 2. Materials and methods

Among the patients who underwent FDG PET/CT imaging for any indication in our institution between July 2021 and December 2021, 129 volunteer patients (M/F: 80/49) who had received mRNA-based COVID-19 vaccine within 42 days were included in this prospective observational study. This research has been approved by the Ethics Committee of Kocaeli University (decision no: GOKAEK-2021/14.18), with the requirement for informed consent waived.

The demographic data, medical diagnosis, treatment history, the date of vaccination, place of vaccination, and previous vaccination history were obtained from all patients before imaging.

Disseminated metastatic disease, ipsilateral breast cancer, or lymphoma that was likely to involve the axilla, and the patients who had ipsilateral intense axillary uptake or thyroiditis on their prior imaging were excluded from the study.

A standard clinical oncologic PET/CT acquisition technique was used for imaging, according to the European Association of Nuclear Medicine guideline, using a PET/CT scanner (GE Healthcare; DISCOVERY 690; acquisition time 2 min per PET bed) [14]. F-18 FDG was intravenously injected into the arm opposite to the previously vaccinated, using a venous line to prevent extravasation. Visual and semiquantitative analyses of patients were evaluated by a nuclear medicine specialist with 12 years of PET/CT experience (SI) and a fellow who had completed 2 years of nuclear medicine training (GK). Maximum standardized uptake values (SUVmax) were recorded with a region of interest drawn on bilateral axillary lymph nodes, healthy bone marrow, spleen, thyroid, and liver for each examination. Deltoid muscle and axillary lymph node (ALN) uptake were considered positive if the ipsilateral/contralateral ALN SUVmax ratio was greater than 1.5 times [15]. The finding of bilateral diffusely increased thyroid uptake with a SUVmax greater than 3.2 was defined as thyroiditis [16,17].

### 2.1. Statistical analysis

All statistical analyses were performed by using IBM SPSS 20.0 (IBM Corp., Armonk, NY, USA). The Kolmogorov-Smirnov test was used to evaluate the normality of distribution for continuous variables. Pearson correlation analysis and Spearman rank correlation analysis were used, respectively, according to whether the variables were normally distributed or not. Categorical variables were expressed as frequency and percentage. Continuous variables were presented as mean ± standard deviation if normally distributed or median and interquartile range (IQR) otherwise. T-test was used for normally distributed and the Mann-Whitney test for nonnormally distributed data to compare continuous variables in the groups and the chi-square test was used to compare categorical variables. For the axillary lymph node (positive/negative), chemotherapy history (with/without), deltoid uptake (positive/negative), and the time interval between vaccination and imaging (0–14 days/>14 days) parameters, binary correlation analyses were performed to evaluate differences. One-way ANOVA or Kruskal-Wallis test was used for comparison among multiple groups (vaccination regimens-one dose, two doses, and heterologous regimen), as applicable. P values less than 0.05 were considered statistically significant.

## 3. Results

Among the patients who underwent FDG PET/CT imaging in our department between July 2021 and December 2021, a total of 129 consecutive patients reported having received an mRNA COVID-19 vaccine dose 6 weeks before imaging, were included in the study. Table 1 summarizes the referral clinics and the FDG PET/CT indication of the patients. Of those vaccinated patients, 47 (36.4%) received heterologous vaccination regimens with the CoronaVac and mRNA booster. The remaining patients received mRNA vaccination regimens. Of those, 36 (27.9%) had one dose, and 46 (35.7%) had two doses of the mRNA vaccine. Overall, 40% (52/129) of vaccinated patients were defined as positive ALN with a median ipsilateral ALN SUVmax of 1.9 (range, 1.5–8.1). Positive ALN was found in 44.7% (21/47) of the patients who received heterologous vaccination regimens (CoronaVac/mRNA), in 44.4% (16/36) of the patients who had one dose, and in 32.6% (15/46) of the patients after two doses of mRNA vaccine. The median age was significantly different between the three groups. The group of patients who had two doses was significantly younger than the patients who received heterologous vaccination regimens and older than the patients with one dose of mRNA vaccine. There was no statistically significant difference in FDG PET/CT variables between the vaccination regimens (Table 2).

Spleen, liver, and bone marrow SUVmax values were statistically higher in the patients with positive ALN, in comparison to patients with negative ALN after mRNA COVID-19 vaccination (Table 3). There was a weak, significant, and negative correlation between ipsilateral ALN SUVmax and the time interval between vaccination and imaging (r = −0.24, p < 0.01) (Figure A). Also, SUVmax of the ipsilateral ALN was weakly but significantly and positively correlated with the SUVmax of spleen, liver, and bone marrow (r = 0.28, p < 0.01, r = 0.20, p = 0.02 and r = 0.20, p = 0.02, respectively) (Figures B–D).

Of all study participants, 32 (24.8%) patients received chemotherapy 5 months before FDG PET/CT (Table 4). The median time from the last day of chemotherapy to FDG PET/CT was 30 days (range, 20–150 days). Positive ALN frequency was 31.3% (10/32) in patients with a history of chemotherapy and 43.3% (42/97) in patients without chemotherapy. The incidence of positive ALN in recently treated patients with chemotherapy was lower than the patients without therapy, but no statistically significant difference was observed.

Positive deltoid muscle uptake was observed in 10 (7.8%) patients. Nine of these patients had positive ALN. The median SUVmax was 2.1 (range, 0.9–8.5) and the median time interval was 4 days (range, 1–6 days) for patients with positive deltoid uptake. Only one patient with deltoid uptake had negative ALN and the time interval between vaccination and imaging was 1 day in this patient. In this study, 32 (24.8%) patients underwent FDG PET/CT imaging in the first week after vaccination and the incidence of deltoid uptake visualization was 31.3% in this subgroup. Additionally, there was a statistically significant relationship between ipsilateral ALN SUVmax, ipsilateral/contralateral SUVmax ratio, positive ALN, time interval, and positive deltoid uptake (Table 5).

Diffuse thyroid uptake on FDG PET/CT was observed in only 8 (6.2%) patients with a median SUVmax of 4.3 (range, 3.5–7.8) and they were defined as thyroiditis. No statistically significant relationship was identified between FDG PET/CT changes, vaccine variables, and patients-defined thyroiditis (p > 0.05).

The time interval between the last mRNA vaccination and FDG imaging ranged from 1 to 42 days (median, 14 days). The median time interval was 9.5 days (range, 1–40 days) for patients with positive ALN and 17 days (range, 1–42 days) for patients with negative ALN. Ipsilateral ALN SUVmax, ipsilateral/contralateral SUVmax ratio values, and the number of patients with positive ALN, and positive deltoid uptake were significantly high in patients who underwent FDG PET/CT within 14 days after mRNA vaccination (Table 6).

Only 8 (6.2%) patients had a positive documented history of COVID-19 infection in our study group who underwent FDG PET/CT imaging. All of these patients were infected before the COVID-19 vaccination. Additionally, no findings of active COVID-19 infection were observed incidentally in the FDG PET/CT images of any of our patients.

## 4. Discussion

Several safe and effective vaccines have been developed to prevent people from getting COVID-19 and to control the pandemic. Due to the immunological effects of vaccines, transient uptake at various sites on FDG PET/CT has been reported after vaccination for different diseases and more recently for COVID-19. Since being more immunogenic than conventional approaches, the mRNA vaccines result in an overestimation of imaging findings. Knowledge of the imaging patterns, pitfalls, and dilemmas of postvaccination FDG PET/CT imaging has become more important than ever in times of COVID-19 [1,2,4–7,18–21]. In addition, there are a few valuable studies about COVID-19 in the Turkish population [22–24].

In the present study, which included patients who received mRNA COVID-19 vaccines manufactured by Pfizer-BioNTech and who underwent FDG PET/CT imaging, we observed positive ipsilateral ALN in 40% of patients suggesting a regional immune response to vaccination, which is consistent with other studies [6,8–12]. The frequency of positive ALN was found in 44.4% of patients who had one dose and in 32.6% with two doses of the mRNA vaccine. The frequency of positive ALN after the first dose was higher than those previously reported [8,9]. This situation can be explained by the fact that patients with one dose are younger than those with two doses as a result of the age-descending vaccination strategies in our country.

After vaccination, increased metabolic activity in the spleen, liver, and bone marrow evaluated by FDG PET/CT, has been reported for other and COVID-19 vaccines [3,13,20,25–28]. In this study, spleen, liver, and bone marrow SUVmax values were statistically high in patients with positive ALN and significantly correlated with ipsilateral ALN SUVmax values. These results indicate that regional metabolic response and systemic immune activation occur together.

In the present study, the frequency of positive ALN was lower in patients with a recent chemotherapy history, but there was no statistically significant difference. Cohen et al. observed similar results and found no association between systemic anticancer therapies and vaccine-associated hypermetabolic ALN [29].

Positive deltoid muscle uptake was found in only 7.8% of our patient population after the first 6 days from vaccination, but 90% of these patients with positive deltoid uptake had positive ALN. Previous studies have also observed the association between deltoid uptake and ipsilateral ALN uptake. In view of these findings, evidence of tracer activity in the vaccination site is a key finding for accurate interpretation of increased FDG activity in ipsilateral ALN [12,13,30,31].

There are several case reports of subacute thyroiditis and Graves’ disease after COVID-19 vaccination, but there is no report of FDG PET/CT findings of thyroiditis after vaccination [32–38]. Diffuse increased thyroid FDG uptake in 6.2% of patients on PET/CT after vaccination was observed. Additionally, no significant difference in the examined clinical variables between patients with and without diffuse thyroid uptake was found.

The frequency of positive ALN on FDG PET/CT in the first two weeks after vaccination was 53% of our cohort. Additionally, ipsilateral ALN SUVmax and incidence of positive ALN showed a decrease with time after vaccination. In this study, the latest positiveness of ALN at 40-day intervals between vaccination and imaging was found. Yael et al. observed the persistence of uptake in ALN on FDG PET/CT 70 days after vaccination [11]. Studies recommend deferring imaging at least 2 weeks after vaccination and may extend the time interval as long as 4 to 6 weeks if can be tolerated, to avoid potentially confusing findings [2,6,8,9,29,39].

The present study has some limitations. The first limitation is the lack of pathological confirmation or follow-up imaging for positive ALN. The second is that in Turkey where this study was conducted, COVID-19 vaccination was started with the elderly age group. Hence, the number of elderly patients and the median age of our cohort is high which caused age heterogeneity. Thirdly, clinical findings and hormonal status were not collected in our patients with diffuse thyroid uptake on FDG PET/CT. Lastly, the titers of antispike antibody concentration at the time of FDG imaging were not examined. It would be beneficial to analyze the relationship between vaccine-related metabolic FDG changes and immune response rate.

## 5. Conclusion

Ipsilateral hypermetabolic ALN is a common regional finding with the most intense FDG uptake in the first two weeks on FDG PET/CT imaging following COVID-19 mRNA-vaccine (Pfizer/BioNTech) administration and gradually decreases within time. Evidence of deltoid uptake in the vaccination site is another early regional finding of the subjects’ images. Furthermore, positive deltoid uptake was observed in 31.3% of patients on FDG PET/CT imaging in the first week of vaccination and this uptake returned to the background level after 6 days in our study. Statistically high metabolic activity in the spleen, liver, and bone marrow was also found in patients with positive ALN, as systemic metabolic changes of vaccines on FDG PET/CT imaging. Our findings suggest that to avoid these transient confusing metabolic changes, it is important to manage the ideal timing of PET/CT imaging or vaccination and get a careful vaccination history.

## Figures and Tables

**Figure:**
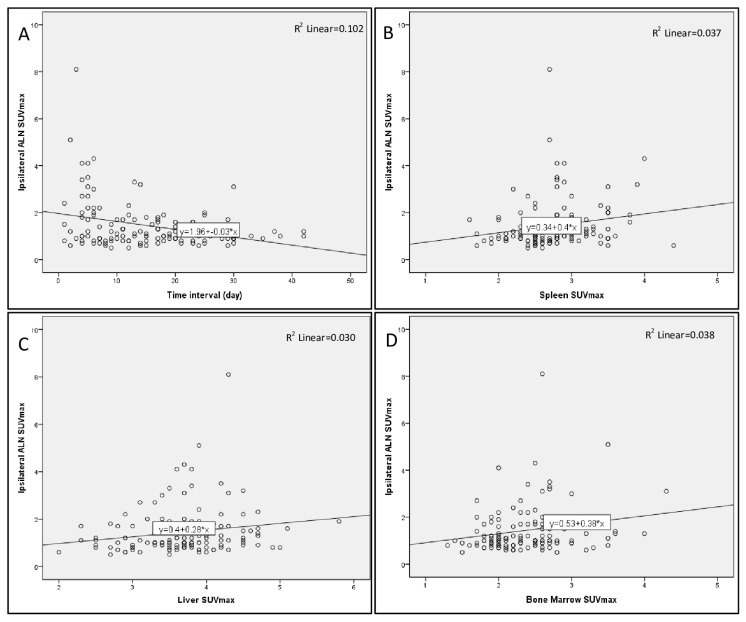
Graphs show a correlation between ipsilateral ALN SUVmax and the time interval between vaccination and imaging (A), spleen SUVmax (B), liver SUVmax (C), and bone marrow SUVmax (D) values.

**Table 1:** The referral clinics and FDG PET/CT indications of the study group.

Characteristic n (%)	Total (n: 129)	One mRNA dose (n: 36)	Two mRNA doses (n: 46)	Heterologous regimen (n: 47)
Primer malignancy				
Lung cancers	39 (30.2%)	9 (25%)	16 (34.8%)	14 (29.8%)
Gastrointestinal cancer	27 (20.9%)	8 (22.2%)	9 (19.6%)	10 (21.3%)
Breast cancer	16 (12.4%)	3 (8.3%)	7 (15.2%)	6 (12.8%)
Lymphoma	6 (4.7%)	5 (13.9%)	0 (0%)	1 (2.1%)
Gynecological cancers	9 (7%)	5 (13.9%)	1 (2.2%)	3 (6.4%)
Genitourinary cancers	5 (3.9%)	3 (8.3%)	1 (2.2%)	1 (2.1%)
Head and neck cancers	14 (10.9%)	0 (0%)	6 (13%)	8 (17%)
Unknown primary	6 (4.7%)	2 (5.6%)	2 (4.3%)	2 (4.3%)
Other	7 (5.4%)	1 (2.8%)	4 (8.7%)	2 (4.3%)
PET/CT indication				
Diagnosis	20 (15.5%)	7 (19.4%)	8 (17.4%)	5 (10.6%)
Staging	45 (34.9%)	11 (30.6%)	19 (41.3%)	15 (31.9%)
Response assessment	32 (24.8%)	8 (22.2%)	9 (19.6%)	15 (31.9%)
Restaging	32 (24.8%)	10 (27.8%)	10 (21.7%)	12 (25.5%)

Values are presented as frequency (percentage).

**Table 2:** The patients’ characteristics and FDG PET/CT changes of vaccination regimens.

Variables	One mRNA dose (n: 36)	Two mRNA doses (n: 46)	Heterologous regimen (n: 47)	P
Age (year)	52 (43–61)	58 (47–64)	62 (54–70)	<0.01
Female (n)	16 (44.4%)	18 (39.1%)	15 (31.9%)	0.5
Interval * (days)	13.5 (5–21)	16 (9–25)	12 (6–23)	0.31
Ipsilateral ALN SUVmax	1.2 (0.9–1.7)	1 (0.9–1.5)	1.1 (0.8–1.9)	0.66
Contralateral ALN SUVmax	0.9 (0.7–1)	0.8 (0.7–1)	0.8 (0.7–0.9)	0.38
SUVmax ratio **	1.3 (1–1.9)	1.2 (1–1.7)	1.3 (1–2.1)	0.62
Spleen SUVmax	2.7 (2.5–3.1)	2.8 (2.5–3)	2.6 (2.5–3)	0.5
Bone marrow SUVmax	2.3 (1.9–2.7)	2.5 (2–2.6)	2.3 (2–2.6)	0.87
Thyroid SUVmax	1.9 (1.8–2.1)	1.9 (1.7–2.4)	2.1 (1.7–2.3)	0.44
Liver SUVmax	3.6 ± 0.7	3.7 ± 0.6	3.7 ± 0.6	0.79
Positive deltoid uptake (n)	5 (13.9%)	2 (4.3%)	3 (6.4%)	0.25
Defined as thyroiditis (n)	0 (0%)	5 (10.9%)	3 (6.4%)	0.13
Received chemotherapy (n)	8 (22.2%)	9 (19.6%)	15 (31.9%)	0.36
Positive ALN (n)	16 (44.4%)	15 (32.6%)	21 (44.7%)	0.42

* The time interval between the last mRNA vaccination and FDG imaging

** Ipsilateral/contralateral ALN SUVmax ratio

Values are presented as frequency (percentage), mean ± standard deviation, or median [interquartile range], as appropriate.

**Table 3:** Characteristics and FDG PET/CT findings of the study population.

Variables	All (n: 129)	Positive ALN (n: 52)	Negative ALN (n: 77)	P
Age (year)	58 (48–65.5)	56 (47.3–63)	58 (49–66.5)	0.28
Female (n)	49 (38%)	23 (44.2%)	26 (33.7%)	0.23
Interval (days)	14 (6.5–23)	9.5 (5–17)	17 (9.5–26)	<0.01
Ipsilateral ALN SUVmax	1.0 (0.9–1.7)	1.9 (1.5–2.7)	0.9 (0.8–1)	<0.01
Contralateral ALN SUVmax	0.8 (0.7–1)	0.8 (0.7–1)	0.8 (0.7–0.9)	0.48
SUVmax ratio	1.2 (1–2)	2.2 (1.8–2.9)	1.1 (1–1.2)	<0.01
Spleen SUVmax	2.7 (2.5–3)	2.8 (2.5–3.2)	2.6 (2.4–2.9)	0.04
Bone marrow SUVmax	2.3 (2–2.7)	2.5 (2.1–2.8)	2.2 (1.9–2.6)	<0.01
Thyroid SUVmax	1.9 (1.7–2.2)	2.1 (1.8–2.4)	1.9 (1.7–2.1)	0.13
Liver SUVmax	3.67 ± 0.64	3.82 ± 0.64	3.57 ± 0.62	0.03
Positive deltoid uptake (n)	10 (7.8%)	9 (17.3%)	1 (1.3%)	<0.01
Defined as thyroiditis (n)	8 (6.2%)	3 (5.8%)	5 (6.5%)	0.87
Received chemotherapy (n)	32 (24.8%)	10 (19.2%)	22 (28.6%)	0.22
One mRNA dose (n)	36 (27.9%)	16 (30.8%)	20 (%26)	0.42
Two mRNA doses (n)	46 (35.7%)	15 (28.8%)	31 (%40.2)	
Heterologous regimens (CoronaVac/mRNA) (n)	47 (36.4%)	21 (40.4%)	26 (%33.8)	

Values are presented as frequency (percentage), mean ± standard deviation, or median [interquartile range], as appropriate.

**Table 4:** Comparison of characteristics and FDG PET/CT findings between patients with and without a history of chemotherapy.

Variables	Received chemotherapy		P
	Yes (n: 32)	No (n: 97)	
Age (year)	61 (43–66)	57 (50–66)	0.85
Female (n)	13 (40.6%)	36 (37.1%)	0.72
Interval (days)	15 (5–28)	14 (7–22)	0.87
Ipsilateral ALN SUVmax	1.1 (0.8–1.7)	1 (0.9–1.7)	0.55
Contralateral ALN SUVmax	0.8 (0.7–1)	0.8 (0.7–1)	0.94
SUVmax ratio	1.2 (1–1.9)	1.3 (1.1–2)	0.40
Spleen SUVmax	2.7 (2.5–3)	2.7 (2.5–3)	0.86
Bone marrow SUVmax	2.2 (2–2.5)	2.4 (2–2.7)	0.11
Thyroid SUVmax	2 (1.8–2.2)	1.9 (1.7–2.2)	0.47
Liver SUVmax	3.6 ± 0.6	3.7 ± 0.6	0.25
Positive deltoid uptake (n)	3 (9.4%)	7 (7.2%)	0.69
Defined as thyroiditis (n)	2 (6.3%)	6 (6.2%)	0.99
Positive ALN (n)	10 (31.3%)	42 (43.3%)	0.22

Values are presented as frequency (percentage), mean ± standard deviation, or median [interquartile range], as appropriate.

**Table 5:** Comparison of characteristics and FDG PET/CT findings between patients with and without deltoid FDG uptake.

Variables	Deltoid uptake		P
	Yes (n = 10)	No (n = 119)	
Age (year)	62 (52–70)	57 (48–65)	0.29
Female (n)	3 (30%)	46 (38.7%)	0.59
Interval (days)	4 (1–5)	15 (9–24)	<0.01
Ipsilateral ALN SUVmax	2.1 (1.8–3.4)	1 (0.9–1.6)	<0.01
Contralateral ALN SUVmax	0.9 (0.7–0.9)	0.8 (0.7–1)	0.98
SUVmax ratio	2.6 (1.8–3.8)	1.2 (1–1.9)	<0.01
Spleen SUVmax	2.8 (2.7–3)	2.7 (2.5–3)	0.27
Bone marrow SUVmax	2.5 (2.2–2.7)	2.3 (2–2.7)	0.18
Thyroid SUVmax	2 (1.9–2.2)	1.9 (1.7–2.2)	0.46
Liver SUVmax	3.8 ± 0.3	3.7 ± 0.7	0.41
Defined as thyroiditis (n)	0 (0%)	8 (6.2%)	0.40
Positive ALN uptake (n)	9 (90%)	43 (36.1%)	<0.01

Values are presented as frequency (percentage), mean ± standard deviation, or median [interquartile range], as appropriate.

**Table 6:** Patients’ characteristics and FDG PET/CT findings in regard to the time interval between vaccination and FDG PET/CT imaging.

Variables	Interval		P
	0–14 days (n = 66)	>14 days (n = 63)	
Age (year)	60 (50**–**69)	56 (46**–**62)	0.13
Female (n)	28 (42.4%)	21 (33.3%)	0.29
Ipsilateral ALN SUVmax	1.2 (0.8**–**2.2)	1 (0.9**–**1.3)	0.04
Contralateral ALN SUVmax	0.8 (0.7**–**0.9)	0.8 (0.7**–**1)	0.26
SUVmax ratio	1.4 (1.1**–**2.5)	1.1 (1**–**1.4)	<0.01
Spleen SUVmax	2.7 (2.5**–**3)	2.7 (2.5**–**3)	0.49
Bone marrow SUVmax	2.3 (2**–**2.6)	2.5 (2**–**2.7)	0.27
Thyroid SUVmax	2 (1.7**–**2.2)	1.9 (1.7**–**2.2)	0.55
Liver SUVmax	3.7 ± 0.6	3.7 ± 0.7	0.97
Defined as thyroiditis (n)	4 (6.1%)	4 (6.3%)	0.95
Positive deltoid uptake (n)	10 (15.2%)	0 (0%)	<0.01
Positive ALN uptake (n)	35 (53%)	17 (27%)	<0.01

Values are presented as frequency (percentage), mean ± standard deviation, or median [interquartile range], as appropriate.
